# Hemorheology and Inflammatory Marker Changes in Patients with Acute Ischemic Stroke after Intravenous Thrombolysis with Mechanical Thrombectomy

**DOI:** 10.12669/pjms.40.3.8396

**Published:** 2024

**Authors:** Li Wu, Peng Shi, Yujie Zhao, Di Shao, Hongliang Wu

**Affiliations:** 1 Li Wu, Department of Neurology, The First Affiliated Hospital of Bengbu Medical College, Bengbu, Anhui Province 233004, P.R. China; 2 Peng Shi, Department of Neurology, The First Affiliated Hospital of Bengbu Medical College, Bengbu, Anhui Province 233004, P.R. China; 3 Yujie Zhao, Department of Neurology, The First Affiliated Hospital of Bengbu Medical College, Bengbu, Anhui Province 233004, P.R. China; 4 Di Shao, Department of Neurology, The First Affiliated Hospital of Bengbu Medical College, Bengbu, Anhui Province 233004, P.R. China; 5 Hongliang Wu, Department of Urology, The First Affiliated Hospital of Bengbu Medical College, Bengbu, Anhui Province 233004, P.R. China

**Keywords:** Acute ischemic stroke, Hemorheology, Inflammatory markers, Intravenous thrombolysis, Mechanical thrombectomy

## Abstract

**Objective:**

To investigate hemorheology and inflammatory marker changes after treatment for acute ischemic stroke (AIS) using intravenous thrombolysis (IVT) with mechanical thrombectomy (MT).

**Methods:**

We retrospectively reviewed clinical records of patients with AIS (*n*=83) treated in The First Affiliated Hospital of Bengbu Medical College between January 2021 and December 2022 (*n*=83). The control group consisted of 38 patients who underwent IVT alone and the observation group consisted of 45 patients who underwent IVT with MT. We compared differences in mean variables related to hemorheology, inflammatory markers, and total efficacy between the two groups.

**Results:**

We found that hemorheology values (plasma viscosity [PV], whole blood viscosity [WBV], fibrinogen [FIB], and hematocrit [HCT]), and the levels of inflammatory markers (tumor necrosis factor ɑ [TNF-ɑ] and interleukin-6 [IL-6]) were higher in the control group than in the observation group after treatment (P<0.05). In addition, the total efficacy of the observation group (93.3%) was higher than that in the control group (76.3%; P=0.016).

**Conclusions:**

The clinical efficacy of combined IVT and MT in the treatment of AIS is superior to IVT alone, improving levels of hemorheology and inflammatory markers in patients with AIS.

## INTRODUCTION

Acute ischemic stroke (AIS) is one of the leading causes of death and disability, caused by insufficient blood supply to the brain due to stenosis or occlusion of the main cerebral vertebral and carotid arteries, and affects about 700,000 people each year.^1^,^2^ Hemorheology refers to flow properties of blood and its cellular components, and patients with AIS have significant baseline hemorheological abnormalities.^3^ Prompt recanalization of affected blood vessels in patients with AIS to prevent irreparable distal tissue damage is of utmost importance.

AIS recanalization treatments (arterial thrombolysis, intravenous thrombolysis [IVT], and mechanical thrombectomy [MT])^3^,^4^ have strict time limits with approximately 75% of blood vessel reopenings occurring within one hour after the onset of symptoms.^1^,^4^ Few vessel reopenings can be attained after the ideal time window, and the disability and mortality rates for patients under such circumstance remain high.^4^ Intravascular mechanical thrombectomy has evolved to become an AIS treatment option with excellent recanalization.^5^,^6^ However, some researchers consider IVT to be a superior first choice to maximize the benefits for the patient and they recommend leaving mechanical thrombolysis as a supplementary treatment.^5^,^6^ We conducted this study to explore the outcomes of patients with AIS undergoing IVT with MT, and we extracted data from the records of 83 patients (treated between October 2021 and October 2022) and compared their hemorheology and inflammatory values as treatment outcome markers after either IVT alone or IVT combined with MT.

## METHODS

We retrospectively analyzed the records of 83 patients with AIS treated at The First Affiliated Hospital of Bengbu Medical College between January 2021 and December 2022. These patients included 42 men and 41 women between the ages of 41 and 76 years (mean, 57.40 ± 7.38 years); 38 patients received IVT (the control group) and 45 received IVT combined with MT (the observation group).

### Inclusion criteria:

- Patients older than 18 years with AIS diagnosed on the basis of imaging examinations;^7^
- Patients presented with AIS for the first time;
- Patients with a time from onset to admission lower than 4.5 hours;
- Patients with complete clinical records.

### Exclusion criteria:

- Patients with a history of intracranial hemorrhage, head injury, or intracranial or intraspinal surgery within the last three months;
- Patients with intracranial tumors, hemorrhage or trauma in other body parts;
- Patients with contraindications to IVT or MT;
- Patients with aortic arch dissection, acute bleeding tendency, severe organ dysfunction, or malignant tumors.

### Ethical Approval

The Medical Ethics Committee of the First Affiliated Hospital of Bengbu Medical College approved this study protocols (2020-219; date, 2020-09-28).

The patients in the control group received IVT alone, and the patients in the observation group received IVT combined with MT.

### IVT procedure

CT and MRI examinations were conducted to detect any hemorrhages and identify embolism sites. Patients received inhaled oxygen to maintain their blood oxygen saturation above 90%, and their glycemic levels were kept between 7.8 and 10 mmol/L. The systolic blood pressures were kept below 180 mmHg and the diastolic blood pressures below 100 mmHg. All patients received thrombolytic therapy with 0.9 mg/kg of tissue type plasminogen activator Alteplase (Boehringer Ingelheim Pharma, S20020034) with a maximum dose 90 mg; 10% of the drug was injected intravenously during 10 minutes, and the remaining dose was diluted in 250 mL of 0.9% normal saline (Shijiazhuang No.4 Pharmaceutical, H20066533) for a one hour IV infusion. The patients underwent brain CT reexamination one day after the thrombolysis to confirm the absence of hemorrhages. Patients were prescribed oral clopidogrel (75 mg/day, Hangzhou Sanofi Company) and aspirin (100 mg/day; Bayer Pharmaceutical Company of Germany) for the first 10 days after the procedure.

### MT procedure

Within eight hours of the thrombolysis procedure, a surgeon inserted a Synchro14 micro-guide wire into the affected blood vessel rotating it through the thrombus. The position of the micro-catheter in the true lumen at the distal end of the obstruction was confirmed with micro-catheter angiography images. After selecting the appropriate Solitaire-FR stent, the surgeon inserted it into the thrombus and opened the stent; which remained in the blood vessel for five minutes before being removed with the thrombus using negative pressure. A second angiography was used to confirm thrombus removal, the procedure was repeated as needed to ensure adequate blood vessel recanalization.

The following hemorheology indicator levels were measured using a Zhongchi fully automatic hemorheology analyzer (ZL6000C): PV, WBV, FIB, and HCT. TAnd, the levels of inflammatory markers TNF-ɑ and IL-6 were automatically obtained with the use of a hemagglutination analyzer (ACL-TOP 700, American Wolfen) performing enzyme-linked immunosorbent assays with American Wolfen reagent. We collected the values obtained before and four weeks after the treatments from the medical records. In addition, we calculated National Institutes of Health Stroke Scale (NIHSS) scores before and three months after treatment, and we assessed the treatment’s efficacy on the basis of the following classification.^8^ We considered patients with an NIHSS score reduction higher than 90% as cured, those with an NIHSS score reduction between 45% and 89% as markedly effective, treatments in patients with score reductions between 17% and 44% as effective, and treatments in patients with score reductions lower than 17% as invalid. We used the following formula to calculate total effective rates:

Total effective rate = (number of cured cases + number of markedly effective cases + number of effective cases)/total number of cases × 100%

### Statistical Analysis

We used SPSS 25.0 (Chicago, IL, USA) for statistical analysis. We expressed continuous variables (age, time from onset to admission, and hemorheology and inflammatory factor indexes) as means ± standard deviations (*χ̅*±S) and compared them applying Student’s *t* or Mann-Whitney’s *U* tests. We expressed categorical variables (gender and cases of curative effect) as number of cases and compared them using Fisher’s exact or *χ^2^* tests. We considered *p-values* < 0.05 as statistically significant.

## RESULTS

We analyzed data from 83 patients with AIS for the study: 38 patients aged between 42 and 74 years (mean, 56.92 ± 7.01 years) underwent IVT (20 men and 18 women) with the median onset-to-admission time of 107.50 min; 45 patients aged between 41 and 76 years (mean, 57.80 ± 7.73 years) underwent IVT combined with MT (22 men and 23 women) with the median onset-to-admission time of 120.00 min. We found similar general data between both groups (*P*>0.05; Table-I) and similar hemorheology and inflammatory marker values for PV, WBV, FIB, HCT, TNF-ɑ, and IL-6 levels between the two groups before treatment (*P*>0.05; Table-II). However, after treatment, all those values in the two groups decreased, with the observation group showed greater improvement than the control group (*P*<0.05; Table-II). The total effective rates were 93.3% for the patients in the observation group and 76.3% for those in the control group (*P*<0.05; Table-III).

**Table-I:** Comparison of general data in the two groups.

Group	n	Gender (male/female)	Age (years)	Time from onset to admission (min)
Control-group	38	20/18	56.92±7.01	107.50 (90.75-150.00)
Observation-group	45	22/23	57.80±7.73	120.00 (94.00-141.50)
*χ^2^*/*t/Z*		0.115	-0.538	-0.443
*P*		0.734	0.592	0.657

**Table-II:** Comparison of hemorheology and inflammatory factor indexes between the two groups (*χ̅*±*S*).

Index		Control-group	Observation-group	t	P
PV (mPa/s)	Before treatment	5.35 ± 0.70	5.40 ± 0.72	-0.312	0.755
	After treatment	3.53 ± 0.55^a^	2.78 ± 0.55^a^	6.209	<0.001
WBV (mPa/s)	Before treatment	7.13 ± 2.02	7.22 ± 2.00	-0.210	0.834
	After treatment	5.31 ± 1.80^a^	3.96 ± 1.68^a^	3.527	<0.001
FIB (g/L)	Before treatment	9.34 ± 1.84	9.51 ± 2.23	-0.380	0.705
	After treatment	4.84 ± 1.54^a^	3.76 ± 1.78^a^	2.932	0.004
HCT (%)	Before treatment	61.37 ± 4.23	60.47 ± 3.99	0.999	0.321
	After treatment	50.58 ± 3.61^a^	37.09 ± 3.66^a^	16.824	<0.001
TNF-ɑ (ng/L)	Before treatment	38.52 ± 3.31	39.71 ± 3.12	-1.675	0.098
	After treatment	16.71 ± 2.64^a^	10.62 ± 2.43^a^	10.924	<0.001
IL-6 (ng/L)	Before treatment	26.79 ± 2.70	26.00 ± 2.22	1.462	0.147
	After treatment	14.79 ± 2.22^a^	8.60 ± 1.79^a^	14.066	<0.001

***Note:***

a P<0.05, compared with values within the group before treatment. PV, plasma viscosity; WBV, whole blood viscosity; FIB, fibrinogen; HCT, hematocrit; TNF-α, tumor necrosis factor α; IL-6, interleukin-6.

**Table-III:** Comparison of total effective rates between the two groups [n (%)].

Group	n	Curative effect				Total efficiency

		Cure	Effective	Valid	Invalid	
Control-group	38	6 (15.8)	12 (31.6)	11 (28.9)	9 (23.7)	29 (76.3)
Observation-group	45	15 (33.3)	21 (46.7)	6 (13.3)	3 (6.7)	42 (93.3)
*χ^2^*	-	-	-	-	-	10.265
*P*	-	-	-	-	-	0.016

## DISCUSSION

We found that IVT combined with MT improved the hemorheology and inflammatory factors of patients with AIS to a larger extent than IVT alone, thereby indicating a superior clinical efficacy of the combination treatment. Huang J et al.^9^ showed that IVT combined with MT promotes vascular recanalization, improves the patient’s neurological function, enhances immune function, and inhibits oxidative stress reactions, while maintaining an appropriate safety profile. Bracard S et al.^10^ conducted a randomized controlled trial in 26 French hospitals and showed that MT combined with IVT can improve the functional independence of patients with acute cerebral ischemia, without increasing their in mortality. Our findings are basically consistent with the studies above.

However, the results of Guimar Rocha M et al.^11^ showed similar intracranial hemorrhage incidences and mortality after either treatment with IVT combined with MT or IVT alone. Choi JH et al.^12^ found similar rates of appropriate functional outcomes for both treatment approaches by comparing clinical and radiological results. Therefore, according to the published evidence, MT alone seems to be a safe and effective treatment strategy for patients who are not eligible for IVT, but the benefits of IVT combined with MT remain unclear.

AIS is caused by different factors leading to reduction or interruption of the blood supply to brain tissues. Initially, ischemic brain tissues present an inflammatory reaction surrounding a central penumbra area with injured neurons that can recover their functionality with prompt reperfusion^13^,^14^, but the brain tissues in severe cases sustaining long ischemic and hypoxic conditions undergo irreversible damage. Thus, successful AIS treatments achieve rapid reperfusion to brain tissues distal to the vessel obstruction.^14^

Studies have shown that patients with AIS display abnormal hemorheology with high levels of PV, WBV, FIB, and HCT that can cause microhemorrhages, thromboses, and vascular endothelial damage aggravation.^15^,^16^ Sung YF et al.^17^ showed that the hemorheology markers of patients with AIS improved significantly after thrombolytic treatment. Moreover, high levels of inflammatory factors such as TNF-ɑ and IL-6 have been associated with AIS development, probably due to the resulting increased adhesion of endothelial and white blood cells and accelerated thrombosis.^18^,^19^ According to Mechtouff L et al.^20^, the levels of TNF-ɑ were abnormally high in patients with AIS before treatment, but decreased significantly after it. Our findings are consistent with those by Lasek-Bal A et al.^21^ indicating that IVT combined with MT is more effective than IVT alone at improving the hemorheology and inflammatory marker levels in patients with AIS. IVT combined with MT leads to quick recanalization of occluded cerebral vessels, restoring blood perfusion to the ischemic brain, reducing the expression of thrombin-antithrombin complexes in plasma, and improving the microcirculation and overall hemorheology values.^21^,^22^

However, MT has been shown to pose a higher risk of complications than drug thrombolysis, such as dissection and perforation.^11^,^23^ International AIS treatment guidelines are not always relevant to Chinese hospitals with different neurosurgery and imaging department capabilities. We believe that both IVT combined with MT and MT have strengths and risks that need to be considered on an individual basis, but regardless of the treatment of choice, physicians need to provide prompt treatments to achieve the best patient outcomes. Patients who meet the indications for IVT and MT should probably undergo this approach to maximize their clinical benefits.

### Limitations of the study

It was a single-center retrospective study with a small sample size, so bias due to patient selection cannot be ruled out. In addition, patients underwent relatively short follow-up periods, with evaluations of few serological and imaging indicators. Moreover, other common AIS treatment methods including simple arterial thrombolysis, simple mechanical thrombolysis, and arteriovenous thrombolysis were not evaluated. Therefore, our results are one-sided, and are conclusions may not be generalizable. Future multi-center, prospective, large-scale studies with long-term follow-up are needed to validate our conclusions.

## CONCLUSION

The clinical efficacy of combined IVT and MT in the treatment of AIS is superior to IVT alone, improving levels of hemorheology and inflammatory markers in patients with AIS.

### Authors’ contributions:

**LW:** Conceived and designed the study.

**PS**, **YZ**, **DS** and **HW:** Collected the data and performed the analysis.

**LW:** Was involved in the writing of the manuscript and is responsible for the integrity of the study.

All authors have read and approved the final manuscript.
